# Copy number gain at 12q12-14 may be important in the transformation from follicular lymphoma to diffuse large B cell lymphoma

**DOI:** 10.1054/bjoc.2000.1638

**Published:** 2001-02

**Authors:** R E Hough, J R Goepel, H E Alcock, B W Hancock, P C Lorigan, D W Hammond

**Affiliations:** Division of Oncology and Cellular Pathology, University of Sheffield, UK

**Keywords:** non-Hodgkin's lymphoma, transformation, comparative genomic hybridization (CGH), 12q12-14

## Abstract

The purpose of this study was to identify novel areas of genomic copy number change associated with transformation from follicular lymphoma (FL) to diffuse large B cell lymphoma (DLBL). DNA was extracted from tumour cells micro-dissected from paraffin- embedded tissue sections in 24 patients with FL and subsequent transformation to DLBL and 18 patients with de novo DLBL. Tumour DNA was compared to reference DNA using comparative genomic hybridization. Abnormalities common to all 3 groups were gains on chromosomes 4q, 5q, 7q, 11q and X and losses on 3p, 8p and 10q. Copy number changes seen in both transformed and de novo DLBL and not seen in FL were gains on 2p and losses on 1q, 15q and Xq. Gains on 2q, 6p, 7p and 17q and losses on 5p and 8q were specific to transformed DLBL cases. Gain on 12q12-14 was found in 52% of the transformed DLBL cases and was never seen in its follicular counterpart. Patterns of genomic copy number change associated with specific clinical events in NHL have been demonstrated and suggest that gains on 2q, 6p, 7p, 12q and 17q and losses on 5p and 8q may be important in the transformation from low to high-grade disease. © 2001 Cancer Research Campaign http://www.bjcancer.com


*British Journal of Cancer* (2001) **84**(4), 499–503
© 2001 Cancer Research Campaign
doi: 10.1054/ bjoc.2000.1638, available online at http://www.idealibrary.com on IDEAL®

http://www.bjcancer.com

### Copy number gain at 12q12-14 may be important in the transformation from follicular lymphoma to diffuse large B cell lymphoma

**RE Hough, JR Goepel, HE Alcock, BW Hancock, PC Lorigan and DW Hammond**

Division of Oncology and Cellular Pathology, University of Sheffield, UK

**Summary** The purpose of this study was to identify novel areas of genomic copy number change associated with transformation from follicular lymphoma (FL) to diffuse large B cell lymphoma (DLBL). DNA was extracted from tumour cells micro-dissected from paraffin-embedded tissue sections in 24 patients with FL and subsequent transformation to DLBL and 18 patients with de novo DLBL. Tumour DNA was compared to reference DNA using comparative genomic hybridization. Abnormalities common to all 3 groups were gains on chromosomes 4q, 5q, 7q, 11q and X and losses on 3p, 8p and 10q. Copy number changes seen in both transformed and de novo DLBL and not seen in FL were gains on 2p and losses on 1q, 15q and Xq. Gains on 2q, 6p, 7p and 17q and losses on 5p and 8q were specific to transformed DLBL cases. Gain on 12q12-14 was found in 52% of the transformed DLBL cases and was never seen in its follicular counterpart. Patterns of genomic copy number change associated with specific clinical events in NHL have been demonstrated and suggest that gains on 2q, 6p, 7p, 12q and 17q and losses on 5p and 8q may be important in the transformation from low to high-grade disease. © 2001 Cancer Research Campaign http://www.bjcancer.com

**Keywords**: non-Hodgkin's lymphoma; transformation; comparative genomic hybridization (CGH); 12q12-14

Follicular lymphoma (FL) accounts for approximately 40% of all non-Hodgkin's lymphoma (NHL). It is an indolent disease with a median survival of 7 to 9 years, but is currently incurable and the majority of patients die of their lymphoma (Bastion et al, 1998). Diffuse large B cell lymphoma (DLBL) constitutes a similar proportion of all cases of NHL. Whilst characterized by an aggressive clinical behaviour, durable remissions can be achieved in up to 50% of patients with combination chemotherapy and radiotherapy (Armitage, 1993). Histological transformation to DLBL will occur in 25–60% of cases of FL (Acker et al, 1983; Horning and Rosenberg, 1984). Progression to DLBL usually coincides with more florid clinical disease which is refractory to treatment and median survival following transformation is less than 12 months (Bastion et al, 1998).

Despite considerable recent advances in our understanding of the cytogenetics and molecular genetics of NHL, the mechanisms responsible for histological transformation and subsequent resistant clinical behaviour remain poorly elucidated. Pathways involving the MYC oncogene (Lee et al, 1989; Yano et al, 1992) or p53 tumour suppressor gene (Lo Coco et al, 1993; Sander et al, 1993) have been implicated.

A clear understanding of the genetic basis for these events will be central to the development of new therapeutic strategies to improve the otherwise poor outcome for this group of patients. We have used comparative genomic hybridization (CGH) to look for novel areas of genomic copy number change in paired biopsies from 24 patients before and after transformation from FL to DLBL and in 18 patients with de novo DLBL.

*Received 3 May 2000*
*Revised 30 August 2000*
*Accepted 18 September 2000*

*Correspondence to:* RE Hough

### MATERIALS AND METHODS

### Patient selection

24 patients with biopsy-proven FL at diagnosis and subsequent transformation to DLBL and 18 patients with de novo DLBL were identified using the regional clinical and pathological databases. Paraffin-embedded tumour tissue from each biopsy was obtained for all patients. The histological diagnoses were confirmed prior to DNA extraction according to the REAL classification by the same expert haematopathologist (JRG) (Harris et al, 1994). Cases of de novo DLBL with remnants of possible FL were noted as these may represent transformation from occult FL. Data regarding presentation, response to treatment, time to transformation and survival were obtained by detailed review of the clinical records.

### DNA extraction

Areas consisting of at least 50% tumour cells were identified by immunocytochemistry using CD20, CD79a, CD43, CD3 and CD45RO. These areas were microdissected from 6–10 serial tissue sections (5 µm) from each biopsy. Genomic DNA was prepared by enzymic digestion using the Qiagen tissue kit. DNA quality was assessed using spectophotometry (Lambda Bio UV/VIS spectrophotometer – Perkin Elmer). The mean of 2 readings at wavelengths of 260 nm and 280 nm were recorded. An estimate of purity was obtained from the ratio of readings at 260 nm and 280 nm (ratios of 1.7–1.9 indicated adequate DNA purity). DNA concentration was calculated from the reading at 260 nm (1OD = 50 µg $ml^{-1}$). DNA of sufficient quality could not be obtained for 6 of the FL biopsies and 1 transformed DLBL biopsy. Once extracted, sample identification was encrypted until completion of all analyses.

### Comparative genomic hybridization

Tumour DNA was compared to reference DNA for copy number change using the technique described by Kallioniemi (Kallioniemi et al, 1992, 1994). Equal amounts (1 μg) of tumour DNA and normal placental, same sex DNA were labelled with Spectrum Green (Vysis) and Spectrum Red (Vysis) respectively by a standard nick translation reaction to give probe fragment length of 300–3000 bp. 800 ng of each labelled DNA and 60 μg of human COT-1 DNA (Gibco) were cohybridized to normal human metaphase chromosomes (Vysis) at 37˚C for 4 days. Chromosomes were counterstained with 4,6-diamidino-2-phenylindole (DAPI) following post-hybridization washes.

### Digital image analysis

3 colour digital images were acquired using a KAF-1400 cooled CCD camera (Photometrics) attached to an epifluorescence microscope (Zeiss Axioskop). Dedicated software, QUIPS (Vysis), was used to calculate the ratio of green (tumour DNA) to red (normal DNA) fluorescence along the length of each chromosome. Mean ratio profiles were calculated using 5 to 8 target metaphases for each biopsy. Abnormalities at 1p32-ter, 16p, 19, 22 and Y were not included as these areas have been found to be unreliable in CGH analysis (Kallioniemi et al, 1994).

### Controls

Ratio values of 1.2 and 0.8 were established as thresholds for gain and loss respectively based on results from 3 controls used with every experiment. These consisted of reference male DNA co-hybridized with reference female DNA, normal female DNA micro-dissected and extracted from paraffin-embedded tonsil co-hybridized with same sex reference DNA and a cell line (MPE 600, Vysis) with established amplifications and deletions against normal opposite sex reference DNA. Ratio values exceeding 1.4 were defined as amplification events.

### Survival

Survival curves were calculated using the method of Kaplan and Meier. The statistical significance of differences observed was determined using the log rank test.

### RESULTS

### Patients

Paired FL and transformed DLBL biopsies were obtained for 24 (9 female, 15 male) patients. Biopsy material of de novo DLBL was obtained for 18 (9 female, 9 male) patients. No previous chemotherapy or radiotherapy had been administered prior to the initial FL or de novo DLBL biopsies with the exception of two patients; one had received radiotherapy and tamoxifen for carcinoma of the breast and subsequently developed FL of the tonsil, the second had achieved a complete remission with PACEBOM chemotherapy for high-grade lymphoma but presented 8 months later with FL.

### CGH data

Copy number gains were seen more frequently than losses (Table 1). The FL biopsies demonstrated the fewest abnormalities. The transformed DLBL had the most complex numerical abnormalities. Figure 1 summarizes gains and losses according to histological subtype; FL, DLBL following transformation and de novo DLBL. A number of abnormalities were common to all 3 types of biopsies (Table 2). Specific patterns of copy number change were seen in the DLBL biopsies irrespective of its origin and were never seen in FL cases (Table 3). A number of abnormalities were only found in the transformed DLBL biopsies that were neither present in the FL counterpart nor the de novo DLBL cases (Table 4). Enh (12q12q14) was seen in 12 of the transformed DLBL biopsies. This abnormality was also found in two de novo DLBL patients; both had histological features suggestive of transformation from an occult FL at histological review prior to this study. One of these patients had a 2 year history of a lump in the right groin and only sought a medical opinion as it suddenly increased in size. The second patient had a clinical presentation and course more typical of de novo DLBL.

**Table 1** CGH data summary

| Mean number of abnormalities (range) | FL[a] | Transformed DLBL[b] | De novo DLBL[b] | All cases |
|---|---|---|---|---|
| Gains | 4.3 (1–9) | 5.5 (1–14) | 4.6 (1–9) | 4.9 (1–14) |
| Losses | 1.2 (0–3) | 3.0 (0–11) | 2.0 (0–9) | 2.2 (0–11) |
| All events | 5.6 (1–12) | 8.5 (4–18) | 6.6 (3–13) | 7.0 (1–18) |
| Chromosomes with gains | 2.7 (1–4) | 3.8 (1–10) | 3.4 (1–8) | 3.4 (1–10) |
| Chromosomes with losses | 1.2 (0–3) | 2.7 (0–8) | 2.0 (0–9) | 2.0 (0–9) |
| Chromosomes with events | 3.8 (1–6) | 6.3 (1–12) | 5.2 (2–11) | 5.2 (1–12) |

[a]FL = follicular lymphoma; [b]DLBL = diffuse large B cell lymphoma.

**Table 2** Abnormalities present in all three groups of biopsies

| Chromosome | Band | Number of FL[a] ($n = 18$) | Number of transformed DLBL[b] ($n = 23$) | Number of de novo DLBL[b] ($n = 18$) | Total ($n = 59$) |
|---|---|---|---|---|---|
| 3p dim | 25–26 | 3 | 3 | 2 | 8 |
| 4q enh | 12–13 | 3 | 4 | 3 | 10 |
| 5q enh | 11.2–13 | 6 | 5 | 4 | 16 |
| 7q enh | 11.2 | 7 | 7 | 2 | 16 |
| 8p dim | 22–23 | 2 | 4 | 7 | 13 |
| 10q dim | 24–26 | 4 | 8 | 2 | 15 |
| 11q enh | 12–13 | 2 | 2 | 3 | 6 |
| Xp enh | – | 10 | 15 | 7 | 32 |
| Xq enh | – | 16 | 18 | 13 | 47 |

[a]FL = follicular lymphoma; [b]DLBL = diffuse large B cell lymphoma.

 © 2001 Cancer Research Campaign

**Table 3** Abnormalities present in both types of DLBL but never found in FCL

| Chromosome | Band | Number of transformed DLBL[a] (*n* = 23) | Number of de novo DLBL[a] (*n* = 18) | Total (*n* = 41) |
|---|---|---|---|---|
| 1q dim | 42–44 | 3 | 2 | 5 |
| 2p enh | 12–16 | 4 | 6 | 10 |
| 12q enh | 12–14 | 12 | 2 | 14 |
| 15q dim | 24–26 | 2 | 2 | 4 |
| Xq dim | 27–28 | 3 | 2 | 5 |

[a]DLBL = diffuse large B cell lymphoma.

High level amplification was detected at 2p12–15 in 1 case, 3p12 (1 case), 11q12-13 (1 case), 12q12-13 (1 case), 12q13-21 (1 case), 18p11.2 (1 case) and on the X chromosome (10 cases).

**Table 4** Abnormalities only present in transformed DLBL

| Chromosome | Band | Number of transformed DLBL[a] (*n* = 23) |
|---|---|---|
| 2q enh | 31–32 | 2 |
| 5p dim | 14–15.3 | 4 |
| 6p enh | 21.3–22 | 2 |
| 7p enh | 11.2–12 | 2 |
| 8q dim | 24.2–24.3 | 4 |
| 17q enh | 11.2–12 | 2 |

[a]DLBL = diffuse large B cell lymphoma.

### Survival

There was a trend towards poorer outcome in FL patients who had gain at 12q12-14 in the transformed DLBL biopsy material; median survival from diagnosis and from transformation were 97 months and 23 months respectively in those who developed

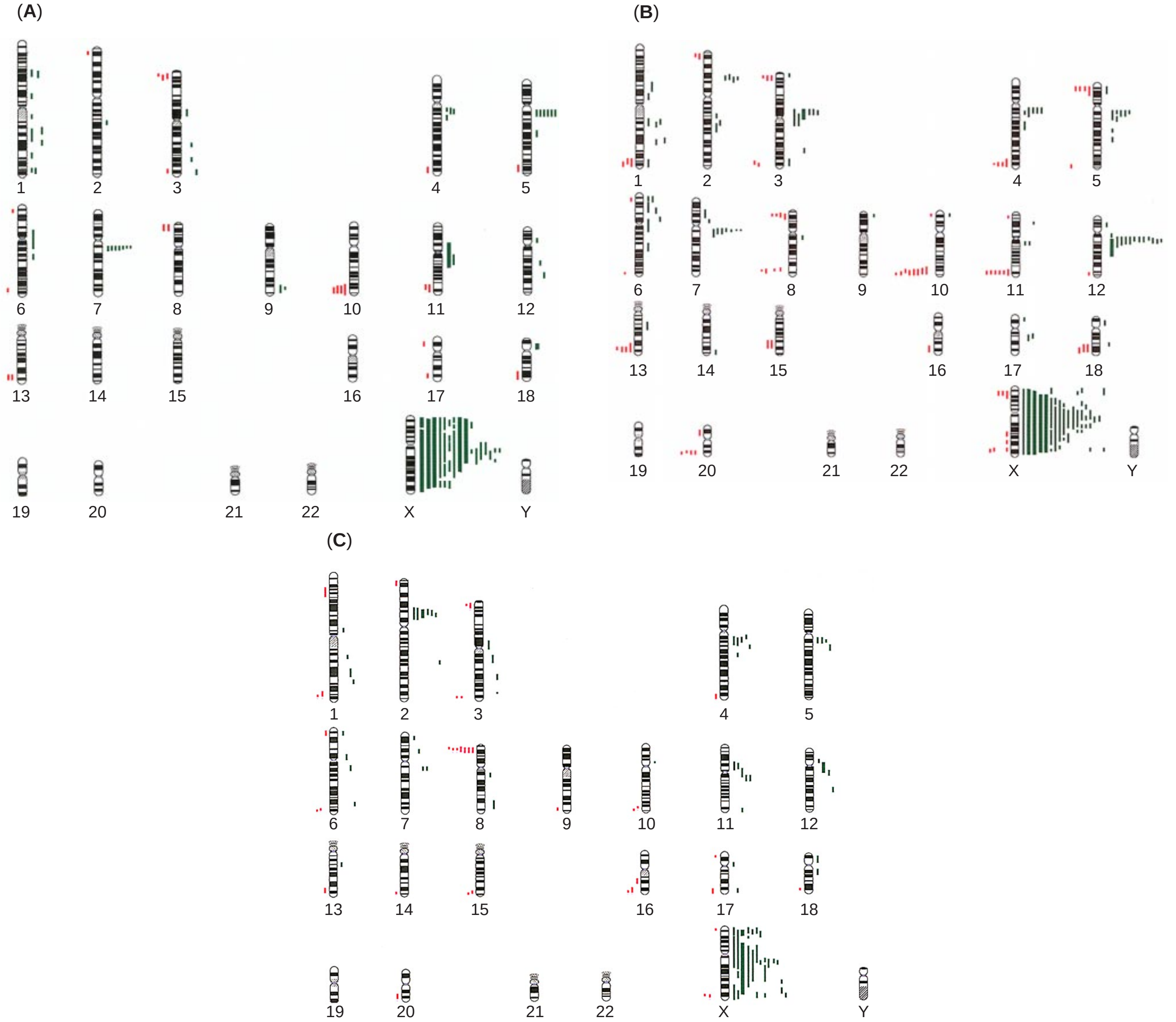

**Figure 1** Ideograms showing cumulative gains and losses on CGH. (**A**) FL biopsies. (**B**) Transformed DLBL biopsies. (**C**) De novo DLBL biopsies. Copy number gains are represented by green lines to the right of each ideogram, whilst copy number losses are represented by red lines to the left. Amplification events are identified by green lines of double thickness.

*© 2001 Cancer Research Campaign*

enh(12q12-14) at transformation and 116 months and 35 months respectively in those who did not. This difference was not statistically significant.

### DISCUSSION

CGH has clearly demonstrated patterns of genomic gains and losses, some of which are common to all groups, with others being specific to histological subtype. The study of NHL by CGH to date suggests that amplification and deletion of oncogenes and tumour suppressor genes respectively may be more important in the pathogenesis of NHL than previously thought (Werner et al, 1997).

Gains of chromosomal material (mean 4.9, range 1–14) were observed more frequently than losses (mean 2.2, range 0–11), a finding consistently demonstrated in all published series of CGH and NHL. The most common abnormalities found, irrespective of subtype, were gains on 4q, 5q, 7q, 11q and X and losses on 3p, 8p and 10q. All subtypes of NHL have developed from the same haematopoietic lineage and it would be reasonable to anticipate certain similarities across the spectrum of this disease. Over-representation on the X chromosome occurs in 21–50% of FL and DLBL in reported CGH series (Bentz et al, 1996; Dierlamm et al, 1996; Joos et al, 1996; Monni et al, 1996; Avet-Loiseau et al, 1997) and was the most common aberration found in this study. This correlates with well established cytogenetic data showing that additional X chromosomes are the most frequent gains in NHL (Fifth International Workshop on Chromosomes in Leukaemia-Lymphoma, 1987; Offit et al, 1991; Hammond et al, 1992), with possible oncogenic sites at Xp22 and Xq28 (Goyns et al, 1993). Losses on 8p and 10q have also been reported in NHL (Goodacre et al, 1994; Avet-Loiseau et al, 1996; Monni et al, 1996) and may represent novel tumour suppressor gene sites.

Comparison of FL and transformed DLBL paired biopsies from the same patient provides a unique picture of the evolution of this disease. Abnormalities found in the FL series were often present in the transformed tissue, supporting the theory that the DLBL has evolved directly from the initial FL clone. The transformed cases were more complex, with a greater number of gains and losses (Table 1). This may be a consequence of the accumulation of genetic aberrations with repeated cell divisions (Yunis et al, 1987) until a critical threshold is reached, beyond which the transformed DLBL develops. However, certain abnormalities were repeatedly found in the transformed tissue that were never present in the FL counterpart, suggesting that specific, non-random events are necessary for transformation to occur.

Gain on 2p and losses on 1q and 15q were present in both the de novo and transformed DLBL biopsies but were never present in FL. High level gain at 2p13-16 has been identified as a common finding in high-grade NHL (Houldsworth et al, 1996; Joos et al, 1996; Werner et al, 1997) resulting from amplification of the *REL* proto-oncogene at this site (Houldsworth et al, 1996; Joos et al, 1996). The specific abnormalities present in the DLBL tissue irrespective of its origin may confer its histological phenotype.

Over-representation on 2q, 6p, 7p and 17q and loss on 5p and 8q were only ever found in the transformed DLBL cases. The histological appearance of de novo and transformed DLBL is usually identical, but the clinical behaviour is quite different. It is possible that the specific genetic abnormalities present in the transformed cases reduce their susceptibility to cytotoxic drugs or ionizing radiation.

Over-representation of 12q12-14 was found in 12 of 23 transformed DLBL biopsies and was never present in the FL counterpart. This gain was also found in 2 of the 18 de novo DLBL cases. Both of these cases had been highlighted in the pathological review at the beginning of the study as having histological features suggestive of transformation from an occult FL. Gain at 12q12-14 is one of the most common abnormalities found in the published CGH data in high-grade NHL (Monni et al, 1996; Joos et al, 1996; Rao et al, 1998). It was not stated in these studies, whether the cases were de novo or transformed high-grade NHL. Emerging data suggest that amplification of an oncogene at this site might also be important in other human tumours (Elkahloun et al, 1996), including sarcoma (Khatib et al, 1993), glioma (Reifenberger et al, 1994), testicular germ cell tumour (Riou et al, 1995), prostatic carcinoma (Sattler et al, 1999) and liposarcoma (Knuutila et al, 1998). The 12q amplicon has been found to be highly complex, exhibiting discontinuous regions of amplification (Wolf et al, 1997). There are a number of candidate genes in this region which include *MDM2, CDK4, CDK2, GL1, ASA*, and *GAD153*. *MDM2* has been shown to be over-expressed in some cases of high-grade NHL (Finnegan et al, 1994). It has a central role in the stabilization of p53 and could theoretically reduce susceptibility to chemotherapy. More recently, a gene encoding the human *BAX* inhibitor, *BI1* has also been identified at this site. When over-expressed in mammalian cells, *BI1* has been shown to suppress *BAX*-induced apoptosis and can interact with *BCL2* which is known to be aberrantly expressed in nearly all FCL (Xu and Reed, 1998).

In contrast to others (Monni et al, 1997; Werner et al, 1997), we found only one case in which there was low-level copy number gain at 18q21, the site of the *BCL-2* gene. The former group, in particular, have frequently found high-level amplification at this locus in DLBL. Werner and colleagues identified two such cases in a series of 62 follicular and diffuse lymphomas (Werner et al, 1997). Such inconsistency may reflect the relatively smaller number of cases in our study or population differences. At other loci, our data are in complete agreement with these studies, with high-level amplifications at 2p and on the X chromosome being frequently observed abnormalities.

If a specific genetic abnormality conferred drug resistance in transformed DLBL, those patients found to have the abnormality would be expected to have a poorer outcome. In this study, a trend towards worse survival was observed in patients with gain at 12q12–14 present in the transformed DLBL DNA (23 months compared with 35 months in those without the aberration). Patients with transformed DLBL have a very poor outcome because the duration of response to currently available treatment modalities is limited. If amplification of a gene contributing to this clinical behaviour could be identified, it would increase our understanding of the molecular mechanisms driving the transformation event and might also provide a target for novel therapeutic strategies of the future.

### ACKNOWLEDGEMENT

This work has been supported by Yorkshire Cancer Research.

### REFERENCES

Acker B, Hoppe RT, Colby TV, Cox RS, Kaplan HS and Rosenberg SA (1983) Histologic conversion in the non-Hodgkin's lymphomas. *J Clin Oncol* **1**: 11–16

© 2001 Cancer Research Campaign

Armitage JO (1993) Treatment of non-Hodgkin's lymphoma. *N Eng J Med* **328**: 1023–1030

Avet-Loisseau H, Mellerin MP, Moreau A, Gaillard F, Vigier M, Harousseau JL and Bataille N (1996) The usefulness of comparative genomic hybridization for analysis of genomic abnormalities in follicular lymphoma: A preliminary study about 28 patients. *Br J Haematol* **93**: 279 (abstr)

Avet-Loiseau H, Vigier M, Moreau A, Mellerin MP, Gaillard F, Harousseau JL, Battaille R and Milpied N (1997) Comparative genomic hybridization detects genomic abnormalities in 80% of follicular lymphomas. *Br J Haematol* **97**: 119–122

Bastion Y, Sebban C, Berger F, Felman P, Salles G, Dumontet C, Bryon PA and Coiffier B (1998) Incidence, predictive factors, and outcome of lymphoma transformation in follicular lymphoma patients. *J Clin Oncol* **15**: 1587–1594

Bentz M, Werner CA, Dohner H, Joos S, Barth TFE, Siebert R, Schroder M, Stilgenbauer S, Fischer K, Moller P and Lichter P (1996) High incidence of chromosomal imbalances and gene amplifications in the classicle follicular variant of follicle centre lymphoma. *Blood* **88**: 1437–1444

Dierlamm J, Rosenberg C, Stul M, Pittaluga S, Michaux L, Wiodarska I, Verhoef G, Thomas J, Janssen M, Bakker-Schut T, Zeller W, Cassiman JJ, Raap A, De Wolf-Peeters C, Van Den Berghe H and Hagemeijer A (1996) Chromosomal gains and losses in marginal zone B-cell lymphoma detected by comparative genomic hybridization. *Blood* **88**: 378a (abstr)

Elkahloun AG, Bittner M, Hoskins K, Gemmill R and Meltzer (1996) Molecular cytogenetic characterisation and physical mapping of 12q13-15 amplification in human cancers. *Genes Chromosom Cancer* **17**: 205–214

Fifth International Workshop on Chromosomes in Leukemia-Lymphoma (1987) Correlation of chromosomal abnormalities with histologic and immunologic characteristics in non-Hodgkin's lymphoma and adult T-cell leukemia-lymphoma. *Blood* **69**: 97–102

Finnegan MCM, Goepel JR, Royds J, Hancock BW and Goyns MH (1994) Elevated levels of MDM-2 and p53 expression are associated with high grade non-Hodgkin's lymphomas. *Cancer Lett* **86**: 215–221

Goodacre A, Ford R and Andreeff M (1994) Comparative genomic hybridization resolves karyotypic heterogeneity in high grade non-Hodgkin's lymphoma. *Blood* **84**: 143a (abstr)

Goyns M, Hammond DW, Harrison CJ, Menasce LP, Ross F and Hancock BW (1993) Structural abnormalities of the X chromosome in non-Hodgkin's lymphomas. *Leukemia* **7**: 848–852

Hammond DW, Goepel JR, Aitken M, Hancock BW, Potter AM and Goyns MH (1992) Cytogenetic analysis of a United Kingdom series of non-Hodgkin's lymphomas. *Cancer Genet Cytogenet* **61**: 31–38

Harris NL, Jaffe ES, Stein H, Banks PM, Chan JKC, Cleary ML, Delsol G, De Wolf-Peeters C, Falini B, Gatter KC, Grogan TM, Isaacson PG, Knowles DM, Mason DY, Muller-Hermelink HK, Pileri SA, Piris MA, Ralfkiaer E and Warnke RA (1994) A revised European-American classification of lymphoid neoplasms: a proposal from the International Lymphoma Study Group. *Blood* **84**: 1361–1392

Horning SJ and Rosenberg SA (1984) The natural history of initially untreated low-grade non-Hodgkin's lymphomas. *N Eng J Med* **311**: 1471–1475

Houldsworth J, Mathew S, Rao PH, Dyomina K, Louie DC, Parsa N, Offit K and Chaganti RSK (1996) REL proto-oncogene is frequently amplified in extranodal diffuse large cell lymphoma. *Blood* **87**: 25–29

Joos S, Otano-Joos MI, Ziegler S, Bruderlein S, Du Manoir S, Bentz M, Moller P and Lichter P (1996) Primary mediastinal (thymic) B-cell lymphoma is characterized by gains of chromosomal material including 9p and amplification of the REL gene. *Blood* **87**: 1571–1578

Kallioniemi A, Kallioniemi OP, Sudar D, Rutovitz D, Gray JW, Waldman F and Pinkel D (1992) Comparative genomic hybridisation for molecular cytogenetic analysis of solid tumours. *Science* **258**: 818–821

Kallioniemi OP, Kallioniemi A, Piper J, Isola J, Waldman FM, Gray JW and Pinkel D (1994) Optimizing comparative genomic hybridization for analysis of DNA sequence copy number change in solid tumours. *Genes Chromosom Cancer* **10**: 231–243

Khatib ZA, Matsushime H, Valentine M, Shapiro DN, Sherr CJ and Look AT (1993) Coamplification of the CDK4 gene with MDM2 and GLI1 in human sarcomas. *Cancer Res* **53**: 5535–5541

Knuutila S, Björkqvist AM, Autio K, Tarkkanen M, Wolf M, Monni O, Szymanska J, Larramendy ML, Tapper J, Pere H, El-Rifai W, Hemmer S, Wasenius VM, Vidgren V and Zhu Y (1998) DNA copy number amplifications in human neoplasms. Review of comparative genomic hybridisation studies. *Am J Pathol* **152**: 1107–1123

Lee JT, Innes DJ and Williams ME (1989) Sequential bcl-2 and c-myc oncogene re-arrangements associated with clinical transformation of non-Hodgkin's lymphoma. *J Clin Invest* **84**: 1454–1459

Lo Coco F, Gaidano G, Louie DC, Offit K, Chaganti RS and Dalla-Favera R (1993) p53 mutations are associated with histologic transformation of follicular lymphoma. *Blood* **82**: 2289–2295

Monni O, Joensuu H, Franssila K and Knuutila S (1996) DNA copy number changes in diffuse large B-cell lymphoma-comparative genomic hybridization study. *Blood* **87**: 5269–5278

Offit K, Jhanwar SC, Ladanyi M, Jilippa DA and Chaganti SRK (1991) Cytogenetic analysis of 434 consecutively ascertained specimens of non-Hodgkin's lymphoma: correlations between recurrent aberrations, histology and exposure to cytotoxic treatment. *Genes Chrom Cancer* **3**: 189–201

Rao PH, Houldsworth J, Dyomina K, Parsa NZ, Cigudosa JC, Louie DC, Popplewell L, Offit K, Jhanwar SC and Chaganti RSK (1998) Chromosomal and gene amplification in diffuse large B cell lymphoma. *Blood* **92**: 234–240

Reifenberger G, Reifenberger J, Ichimura K, Meltzer PS and Collins VP (1994) Amplification of multiple genes from chromosomal region 12q13–14 in human malignant gliomas: Preliminary mapping of the amplicons shows preferential involvement of CDK4, SAS, and MDM2. *Cancer Res* **54**: 4299–4303

Riou G, Barrois M, Prost S, Terrier M, Theodore C and Levine AJ (1995) The p53 and MDM2 genes in human testicular germ cell tumours. *Mol Carcinogen* **12**: 124–131

Sander C, Yano T, Clark HM, Harris C, Longo DL, Jaffe ES and Raffeld M (1993) p53 mutation is associated with progression in follicular lymphomas. *Blood* **82**: 1994–2004

Sattler HP, Zimmer E, Wellman A, Rohde V and Wullich B (1999) Amplification of growth regulatory genes on 3q25–q27 in human prostate cancer. *Proc AACR* **40**: 542 (abstr)

Werner CA, Dohhner H, Joos S, Trumper LH, Baudis M, Barth TFE, Ott G, Moller P, Lichter P and Bentz M (1997) High level DNA amplifications are common genetic aberrations in B-cell neoplasms. *Am J Pathol* **151**: 335–342

Wolf M, Aaltonen LA, Szymanska J, Tarkkanen M, Blomqvist C, Berner JM, Myklebost O and Knuutila S (1997) Complexity of 12q13–22 amplicon in liposarcoma: microsatellite repeat analysis. *Genes Chrom Cancer* **18**: 66–70

Xu Q and Reed JC (1998) Bax inhibitor-1, a mammalian apoptosis suppressor identified by functional screening in yeast. *Molec Cell* **1**: 337–346

Yano T, Jaffe ES, Longo DL and Raffeld M (1992) MYC rearrangements in histologically progressed follicular lymphomas. *Blood* **80**: 758–767

Yunis JJ, Frizzera MM, Oken J, McKenna A, Theologides A and Arsnesen M (1987) Multiple recurrent genomic defects in follicular lymphoma. A possible model for cancer. *N Eng J Med* **316**: 79–84

© 2001 Cancer Research Campaign 
